# Supplementary material for: Spectroelectrochemical analysis of the mechanism of (photo)electrochemical hydrogen evolution at a catalytic interface
Source: Nat Commun. 2017 Feb 24;8:14280. doi: 10.1038/ncomms14280 (PMC5333116; doi:10.1038/ncomms14280)
Supplement: Supplementary Information — Supplementary Figures [file ncomms14280-s1.pdf]

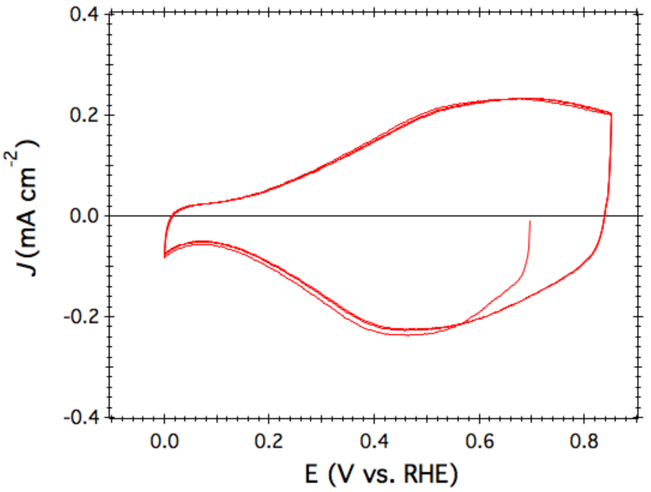

Supplementary Figure 1: Cyclic voltammogram of the RuO_x catalytic layer measured at a scan rate of 50 mV s^{-1}.

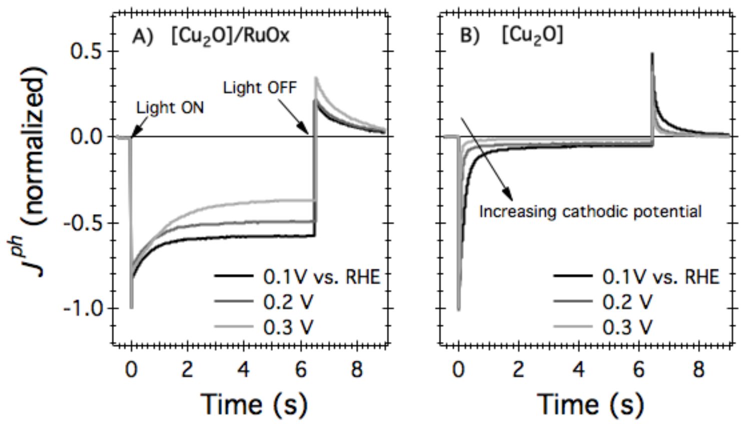

Supplementary Figure 2: Normalized photocurrent transients of the [Cu_2O] photocathode (A) with a RuO_x catalytic layer and (B) without the RuO_x. The current response is measured following a 6 s illumination pulse (365 nm light) at applied potentials ranging from +0.3 to 0.1 V vs. RHE

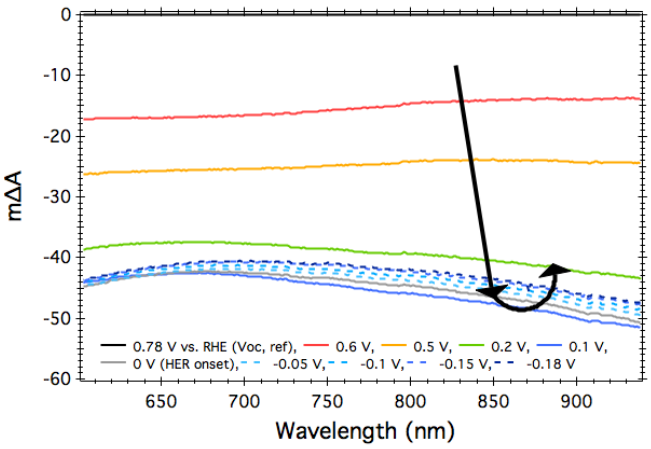

Supplementary Figure 3: Change in absorbance of the RuO_x HER catalyst as a function of the applied potential. The spectroelectrochemical changes in absorbance were obtained representing the absorbance against the absorbance of the catalyst at its OCP (0.78 V vs. RHE).

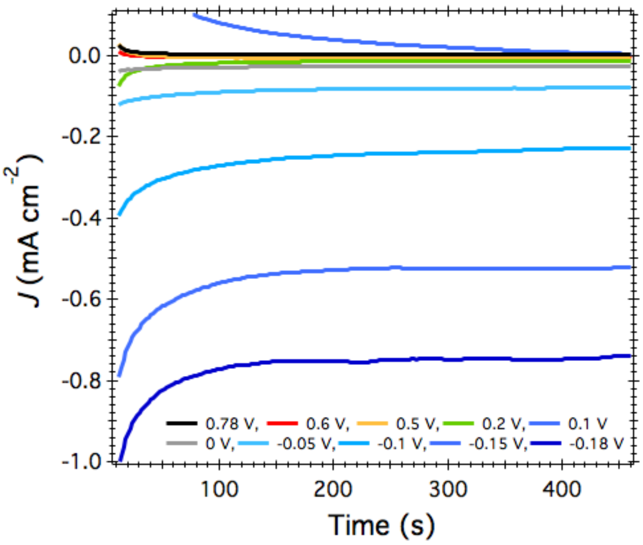

Supplementary Figure 4: Current-Time characteristics of the RuO_x HER catalyst as a function of the applied potential.

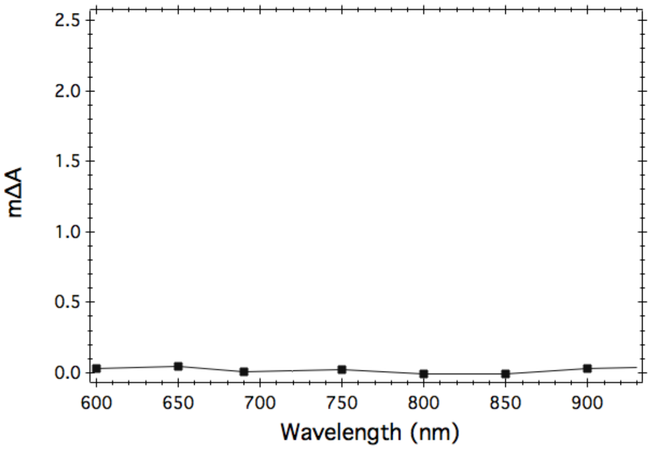

Supplementary Figure 5: Photoinduced changes in absorbance obtained by measuring the steady state change in absorbance of a TiO_2/RuO_x film attained upon ~ 5-6 s, 365 nm illumination.

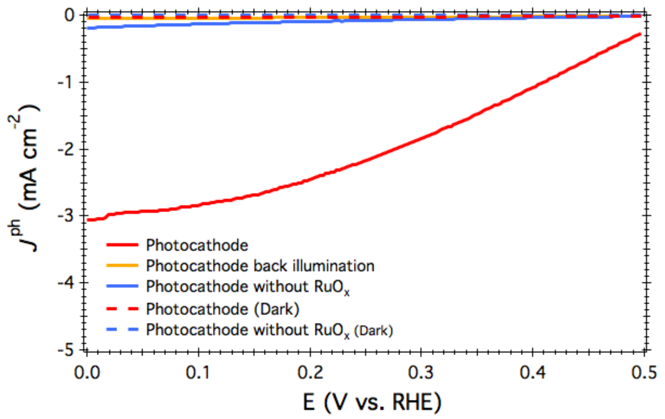

Supplementary Figure 6: Current-Potential characteristics of the [Cu_2O] photocathode in the dark (dashed lines) and under conditions of front and back illumination (solid lines) employing 365 nm light (~40mW cm^{-2}), with and without the RuO_x catalytic layer.

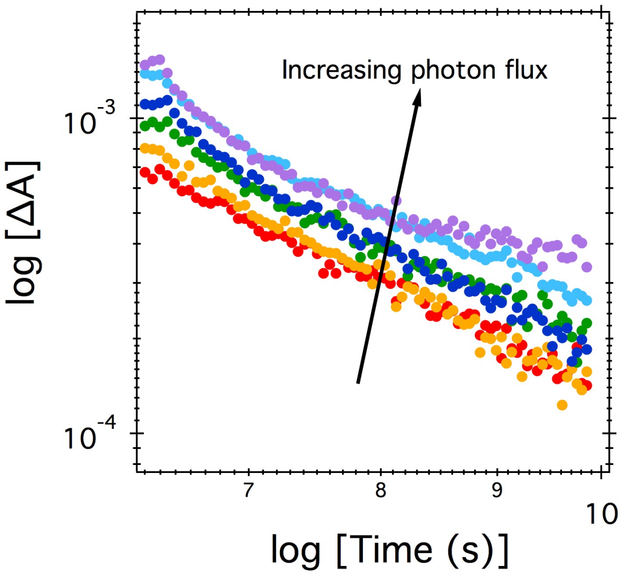

Supplementary Figure 7: Photoinduced absorption changes of the [Cu_2O]/RuO_x photocathode following light off assigned primarily to the decay of RuO_x(2-) due to the reduction of protons. Full dataset (light on and light off) shown in Figure 5.

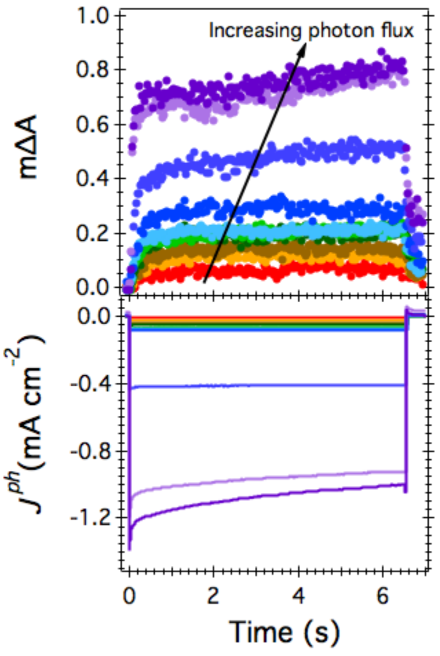

Supplementary Figure 8: Photoinduced absorption changes of the [Cu_2O]/RuO_x photocathode in a solution containing 0.01 M surfactant (Tritron X-100), used to promote facile bubble release, upon 365 nm light illumination at different photon fluxes at a fixed bias of 0.1 V_{RHE} and the photocurrent measured simultaneously.
